# Macro-Particle Charcoal C Content following Prescribed Burning in a Mixed-Conifer Forest, Sierra Nevada, California

**DOI:** 10.1371/journal.pone.0135014

**Published:** 2015-08-10

**Authors:** Morgan L. Wiechmann, Matthew D. Hurteau, Jason P. Kaye, Jessica R. Miesel

**Affiliations:** 1 IGDP in Ecology, The Pennsylvania State University, University Park, Pennsylvania, United States of America; 2 Department of Ecosystem Science and Management, The Pennsylvania State University, University Park, Pennsylvania, United States of America; 3 Department of Forestry, Michigan State University, East Lansing, Michigan, United States of America; Chinese Academy of Sciences, CHINA

## Abstract

Fire suppression and changing climate have resulted in increased large wildfire frequency and severity in the western United States, causing carbon cycle impacts. Forest thinning and prescribed burning reduce high-severity fire risk, but require removal of biomass and emissions of carbon from burning. During each fire a fraction of the burning vegetation and soil organic matter is converted into charcoal, a relatively stable carbon form. We sought to quantify the effects of pre-fire fuel load and type on charcoal carbon produced by biomass combusted in a prescribed burn under different thinning treatments and to identify more easily measured predictors of charcoal carbon mass in a historically frequent-fire mixed-conifer forest. We hypothesized that charcoal carbon produced from coarse woody debris (CWD) during prescribed burning would be greater than that produced from fine woody debris (FWD). We visually quantified post-treatment charcoal carbon content in the O-horizon and the A-horizon beneath CWD (> 30 cm diameter) and up to 60 cm from CWD that was present prior to treatment. We found no difference in the size of charcoal carbon pools from CWD (treatment means ranged from 0.3–2.0 g m^-2^ of A-horizon and 0.0–1.7 g m^-2^ of O-horizon charcoal) and FWD (treatment means ranged from 0.2–1.7 g m^-2^ of A-horizon and 0.0–1.5 g m^-2^ of O-horizon charcoal). We also compared treatments and found that the burn-only, understory-thin and burn, and overstory-thin and burn treatments had significantly more charcoal carbon than the control. Charcoal carbon represented 0.29% of total ecosystem carbon. We found that char mass on CWD was an important predictor of charcoal carbon mass, but only explained 18–35% of the variation. Our results help improve our understanding of the effects forest restoration treatments have on ecosystem carbon by providing additional information about charcoal carbon content.

## Introduction

Changing climate is projected to result in regional warming and drying in the western United States [1–3], increasing large wildfire frequency across the region [4–6]. Additionally, 20^th^ century fire suppression has increased tree density and the accumulation of forest floor biomass, making forests that evolved with a high-frequency, low-severity fire regime more prone to high-severity, stand-replacing fire events [7,8].

Fire emissions from these wildfire events represent a large and highly variable component of the United States carbon (C) budget [9]. In the western United States, forests can remain a net source of C for years to decades following a stand replacing wildfire event [10,11]. Because forests are valued globally for mitigating atmospheric carbon dioxide, concern about climate change has made mitigation of greenhouse gas emissions and increased C storage a priority for forest managers [12].

In dry, frequent-fire forests, altering forest structure and decreasing fuel loads through mechanical thinning and prescribed burning can increase forest resistance to high-severity, stand-replacing wildfires [13]. By reducing the risk of stand-replacing wildfires, forest managers may also be protecting against potential C losses by reducing direct wildfire C emissions and increasing resistance to ecosystem switching following the wildfire event (e.g. to grasslands or shrublands) [14,15]. This is assuming that the C losses associated with the management practices are less than the avoided C losses associated with the wildfire or alternate vegetation state.

Understanding the influence that prescribed fire has on the rates of C accumulation or loss is essential for quantifying total ecosystem C flux to the atmosphere, and thus accurate C accounting. Various studies have investigated forest C losses from restoring fire and forest structure [15–20], but few studies have investigated the potential long-term C storage resulting from prescribed fire [21, 22]. Fire may contribute to long-term C storage by producing pyrogenic carbon (hereafter, charcoal), a potentially long-term C pool that has often been overlooked [21,23].

Charcoal is formed by thermal decomposition during the incomplete combustion of biomass. Charcoal is one component of the black C continuum, which ranges from partly charred plant material to soot and graphite particles [24,25]. Relative to other C pools in the soil, charcoal is C-rich and nitrogen-depleted, and has a highly aromatic molecular structure that may contribute to increased resistance to microbial degradation. These characteristics make charcoal a relatively stable soil C component [24], with particles dating more than 10,000 years back in time [26,27]. Although charcoal is not inert in the environment, it is among the most slowly-cycling forms of C [28], with residence times that indicate that it may be an important contributor to global C sequestration in soils [29–31]. Charcoal may also influence C cycling in forest ecosystems. For example, anthropogenic charcoal such as biochar has been used to improve soil fertility by lowering the soil pH [32], increase soil microbial biomass [33], and improve nutrient retention through cation adsorption [34]. These properties ultimately influence plant growth and C cycling.

Little direct field evidence has been collected to quantify soil charcoal content in forests that experience low-severity fire, such as prescribed burning (except see [35–37]). DeLuca and Aplet [21] suggested that fuel reduction treatments that do not include prescribed burning, the disturbance process that produces charcoal, may reduce soil charcoal content due to erosion and thus, potential long-term C storage in mineral soils. After a prescribed fire in a Florida scrub-oak forest, approximately 25% (140 g C m^-2^) of litter-fall was converted into charcoal [35]. In a southeastern US conifer forest charcoal production rates ranged from 0.38–1.53% of available biomass and in a northwestern US conifer forest production rates ranged from 1–8% of woody fuels [36, 38]. In conifer forests of the western US, potential combustible biomass on the ground such as coarse woody debris (CWD) may provide a source for charcoal. In Sierra Nevada mixed-conifer forests, CWD represents 3–8% of total ecosystem C, storing 12–27 Mg C ha^-1^, with spatial distributions ranging from significantly clustered to random [39–41]. Without directly quantifying potential charcoal C additions from incompletely combusted CWD during prescribed burning, we may be underestimating a long-term C pool of charcoal. Furthermore, even if charcoal formation rates are low, the relatively long residence time of charcoal in soil means that even small changes in inputs could have large impacts on long-term soil C storage [21].

The purpose of this study was to quantify the effects of pre-fire fuel load (biomass on forest floor, Mg ha^-1^) and fuel type (CWD or FWD) on charcoal C content resulting from different fuel reduction treatments that included prescribed burning in a mixed-conifer forest. We tested the following hypotheses: (1) charcoal C content is greater from the incomplete combustion of CWD than the incomplete combustion of smaller fuels, (2) burned forest stands would contain more soil charcoal C than forest stands that were not burned, and (3) thinned and burned treatments would have more charcoal C than treatments that were only burned, because of the large inputs of CWD from thinning.

## Materials and Methods

### Study Site

This study was conducted in an old-growth mixed-conifer forest within the Teakettle Experimental forest, a 1300 ha reserve located approximately 80 km east of Fresno, California (USA). The US Forest Service whom provided permission for this research manages the Teakettle Experimental forest. This study did not involve any endangered or protected species. The climate is characterized as Mediterranean, with average annual precipitation of 125 cm, falling almost exclusively as snow [42]. The elevation ranges from 1900–2600 m. This mixed-conifer forest ecosystem is dominated by five tree species: *Abies concolor*, *A*. *magnifica*, *Calocedrus decurrens*, *Pinus jeffreyi*, and *P*. *lambertiana*. The soils within the treatment units are Inceptisols and Entisols from the Cagwin (pH 5.4) and Cannell (pH 6.0) series. Both series are granitic soils that are highly permeable and have coarse sandy loam texture throughout the profile. The Teakettle experiment, located in the Experimental Forest, was established to examine the ecological effects of a range of structural manipulations and prescribed burning. The experiment utilized a full factorial design, crossing three levels of thinning (no thin, understory-thin, overstory-thin) with two levels of burning (no burn, prescribed fire). Three replicates of each treatment were established using four-hectare treatment units. The understory-thin treatment removed all trees 25–75 cm diameter at breast height (DBH). The understory-thin was initially designed to reduce impacts on California spotted owl (*Strix occidentalis occidentalis*) habitat, although the guidelines now have been primarily used to reduce stand-replacing fire risk in Sierra mixed-conifer forests. The overstory treatment removed all trees greater than 25 cm DBH, with the exception of 22 large diameter trees ha^-1^. The thin and burn plots were mechanically treated in 2000 and burned in 2001. Prescribed fires were implemented during fall 2001 when mean daytime temperature was 13°C and relative humidity ranged from 25–70% throughout the day [43]. The fires burned 20–40% of the surface area in unthinned plots and 35–70% of thinned plots [43].

### Previous Data Collection

As part of the larger Teakettle experiment, prior to treatment implementation (1998–2000), all trees within the 4 ha treatment units were mapped with a surveyor’s total station, tagged, and measured for species, height, condition class, and decay class for dead trees [42]. Within each treatment unit, 9 monumented gridpoints were established on 50 m grid in two of the three replicates and 49 monumented gridpoints were established in one of the replicates on a 25 m grid. At each gridpoint 10 m^2^ circular plots were established to measure understory vegetation, and tree regeneration [42]. Three 15 m modified Brown’s planar intercept transects were established at each gridpoint to measure litter depth and fine woody debris (FWD; diameter 5–9 cm) [44]. Following treatment implementation (2002–2004), the experimental units were re-sampled following the same protocols. A complete post-treatment CWD inventory was collected and included mapping each piece and measuring end diameters, length, decay class, and species [39]. Additional measurements within the circular plots at the gridpoints of the burn treatments included visually estimating percent ash cover (denoted as 2002% ash in tables) on the forest floor and percent char on all coarse woody debris (CWD) falling within the circular plots (denoted as 2002% CWD charred in tables and figures) [45]. We used these additional measurements as parameters in our model selection procedure for estimating charcoal pools.

### Field Sampling

We focused our sampling efforts on charcoal formed from coarse woody debris (CWD: dead woody material ≥ 30 cm diameter; hereafter, log) during the 2001 prescribed fire. Sampled logs were chosen from a pre-existing database that included logs that were mapped during the complete CWD inventory. We used this spatially explicit database of log measurements to identify individual logs that were within 15 m of the monumented grid points to coincide with the length of the FWD inventory transects. Previous research found no statistical differences in FWD between treatments that included burning [38] and a within treatment unit comparison of FWD inventory transects did not detect significant FWD loading differences between gridpoints within treatment units. From the candidate population of all mapped logs, we randomly selected 100 individual logs (10 logs from each treatment unit that included burning and 10 from the control units). We collected 14, 10.16 cm-diameter samples at each log, including seven samples of the full depth of the organic horizon (Ohor) and seven of the top 5 cm of the A horizon (Ahor). Three samples were taken directly adjacent to the log using 50 cm spacing on the down slope side of the log. Four additional samples were collected using a transect that ran perpendicular to the log for 60 cm from the mid-point (S1 Fig). Off-log samples were spaced at 5 cm, 15 cm, 30 cm, and 60 cm down slope of the log. Prior to the prescribed burn, the last surface fire at the site was in 1865 [46]. In an effort to limit sampling to charcoal particles formed during the 2001 prescribed fire, we constrained the A horizon samples to 5 cm in depth. The organic horizon was removed and separated prior to collecting the A horizon. While the Ahor was consistently sampled to 5cm depth, total sample depth (Ohor + Ahor) varied as a function of organic horizon thickness. We also measured depth of charring into woody tissue on each log at three locations and calculated char mass on each log following Donato et al. [23] to obtain an estimate of total char produced during the 2001 prescribed burn, (denoted CWD charred mass in tables and figures). Depth of char was measured at random locations on the sides and tops of logs. We used assumptions from Donato et al. [23] in our calculations that included 70% mass loss with charring and mean wood density of 0.2735 g cm^-3^, regardless of decay class because we measured char 12 years after the prescribed fire. The size of the logs precluded sampling on the bottoms. We collected data on char mass on each log to use as a predictor variable of charcoal macro-particle C in the Ohor and Ahor.

### Lab Analysis

We used 1 mm and 2 mm sieves to isolate and collect charcoal macro-particles from a subset (n = 287 A hor and n = 247 O hor) of the 700 total subsamples for each horizon to determine the C contribution of each size class. Because the 2 mm sieve captured the majority of charcoal macro-particle C (Table 1), we only sampled for charcoal particles > 2 mm in the remainder of the subsamples. While this approach captured 92% of the macro-particle C in the A horizon and 86% of the macro-particle C in the organic horizon, other separation methods have found that the <0.5 mm fraction accounted for 23.5% of total charcoal C [47]. Following methods similar to Brimmer [48] and Alexis *et al*. [35], charcoal separation was performed on white trays under supplemental light using the unaided eye for the 2 mm size class and magnifying lamp with an enlargement factor of 175% for the 1 mm size class.

**Table 1:** Mean and (standard error) of charcoal carbon (C) and the C concentration in two particle size classes within the O horizon per 10.16 cm core.

Treatment (sample size)	Size Class	Mean Charcoal mass (g)	Carbon Concentration (%)	Mean Charcoal C mass (g)
Control (44)	>2 mm	0.0 (0.0)	15 (0.0)	0.0 (0.0)
	1–2 mm	0.0 (0.0)	29 (0.0)	0.0 (0.0)
Burn Only (50)	>2 mm	1.5 (0.5)	58 (0.0)	1.0 (0.3)
	1–2 mm	0.2 (0.1)	47 (0.0)	0.1 (0.0)
Understory thin/burn (109)	>2 mm	1.3 (0.3)	59 (0.0)	0.9 (0.2)
	1–2 mm	0.1 (0.0)	46 (0.0)	0.1 (0.0)
Overstory thin/burn (84)	>2 mm	1.0 (0.0)	54 (0.0)	0.7 (0.0)
	1–2 mm	0.1 (0.0)	42 (0.0)	0.0 (0.0)

After charcoal was removed from the O and A horizons, the charcoal from each sample was dried at 65°C, weighed and ground. We used an EA 1110 CHNS-O (CE Instruments 1996) elemental analyzer to obtain the percent C and nitrogen (N) of the charcoal from all charcoal samples. We also quantified percent hydrogen in the charcoal_Ahor_ using the same elemental analyzer. We measured percent ash on a subset of charcoal samples following ASTM E1755-01 [49] and we used the mean ash weight percent to calculate oxygen concentration by difference [47].

### Data analysis

To test the hypothesis that more charcoal is produced from CWD than FWD, we used a linear regression analysis to compare charcoal mass (g) from samples collected adjacent to the log to the mass of charcoal collected at the varying distances away from the log. Charcoal_Ohor_ and charcoal_Ahor_ were analyzed separately.

A nested analysis of variance (ANOVA) was used to compare treatment effects on charcoal C pools at logs. Charcoal C samples from each log were averaged and converted to a per unit area measure. Total C concentrations were scaled to an area basis (g m^-2^) using the volume of organic matter and mineral soil samples, data on log length, the perpendicular transect length (60 cm), and previously derived bulk density (BD) measurements at Teakettle experimental forest (A horizon BD: 0.95 (se = 0.02) g cm^-3^ for 0–10 cm, organic horizon range: 0.10 (se = 0.01) to 0.11 (se = 0.01) g cm^-3^). Our sampling unit was the log (n = 30 for treatments that burned, n = 10 for un-burned controls) and we treated soil cores as subsamples within the sampling unit. Data were log-transformed to meet all assumptions for the ANOVA. We used Tukey’s HSD post hoc analysis to determine if there were significant (*p* < 0.05) differences between treatments.

To estimate Ohor and Ahor (top 5 cm) charcoal pools in each treatment unit we obtained information on log length for all logs in the treatment units using the pre-treatment log dataset. We estimated charcoal C from the area adjacent to each log in the O horizon by using the mean depth and for the top 5 cm of the A horizon. The area adjacent to each log was the length of the sampled log multiplied by the transect length (60cm). We used mean charcoal C values from the log sampling area by treatment to estimate the pool of charcoal C in each four hectare treatment unit (Mg charcoal C ha^-1^). We used ANOVA to compare charcoal C pools among the different treatments at the hectare scale (n = 3). Based on this estimate we were able to compare the size of Ohor and Ahor (top 5 cm) charcoal C pools with other forest C pools (e.g. total soil, live tree, etc) on a per hectare basis. Data were log-transformed to meet the ANOVA assumptions of normality and equal variance and Tukey’s HSD post hoc analysis was used to determine if there were significant (*p* < 0.05) differences between treatments.

To quantify the effect of prescribed fire on charcoal C content, we used linear regression models in an information theoretic framework [50]. The variables included in the analysis were pre-treatment fine woody debris (Mg C ha^-1^); pre-treatment litter depth (cm), 2002 visually estimated percent coarse woody debris charred, 2002 percent ash cover, and 2013 char mass on sampled CWD (Mg C). From these predictor variables we developed a candidate set of models that included charcoal C (g C m^2^) as the response variable. We ranked all possible model combinations for each treatment using the Akaike Information Criterion for small samples (AIC_C_) [50]. We chose the best model (lowest AIC_C_ value) to estimate charcoal C as a function of the fire effect predictors for each treatment (burn-only, understory-thin and burn, and overstory-thin and burn). Data were log-transformed to meet the assumptions of normality and equal variance.

## Results and Discussion

Changing climatic conditions and a century of fire exclusion have increased the risk of large stand-replacing wildfire events in Sierran forests, a trend that is projected to continue [6,51–53]. Large wildfire events release substantial amounts of C to the atmosphere and with projected increases in large wildfire frequency; emissions from fire are projected to increase from the most C dense forested areas of California [54]. Previous research has demonstrated that restoring natural fire regimes to forests that were historically adapted to frequent fire, such as Sierran mixed-conifer forests, reduces the risk of stand-replacing wildfire, but is not without costs in terms of C emissions to the atmosphere [55]. We sought to better quantify the C costs and benefits associated with prescribe fire (i.e. restoring natural fire regimes) by estimating charcoal C pools formed from incompletely combusted ground woody debris. Charcoal C may be an important C pool because of its worldwide distribution in soils and chemical properties that contribute to a long mean residence time in the environment relative to other forms of organic matter [56].

### Charcoal chemical properties

The charcoal quantities obtained in this study most likely represent a conservative estimate of macroscopic charcoal produced from a prescribed fire because we confined our sampling to particle sizes greater than 2 mm. However, our subsampling for smaller macro-particles suggest that we captured the majority (86% (se = 1.7) in the O horizon, and 92% (se = 0.5) in the A horizon) of the macroscopic charcoal C produced during the burn treatments. Over time, the larger particles may succumb to physical breakdown and eventually contribute to smaller particle size classes. However, we also found charcoal in the control treatments that have not experienced fire since 1865 [46], demonstrating that macroscopic charcoal from past fires can persist for at least 148 years. We emphasize that our estimates of charcoal in forest soil are based on visually identified char rather than methods that estimate or characterize fire-affected organic matter in bulk soil via chemical oxidation (e.g., [57, 58]). Charcoal C concentrations were similar (50–60%) among treatments and between soil horizons (charcoal_Ohor_ and charcoal_Ahor_), with the exception of charcoal_Ohor_ C concentration in the control (15% C) (Table 2). The H/C ratio was similar across all treatments (Table 2). Charcoal C represented a small fraction of total soil organic carbon (SOC), ranging from 0.94–1.48% (Table 2).

**Table 2:** Mean and (standard error) of charcoal mass, C concentration, and charcoal C mass in the two particle size classes within the top 5 cm of the A horizon per 10.16 cm soil core. Also, the percent (%) charcoal C of total soil carbon for combined A and O horizons. Values in the parentheses are standard errors of the means.

Treatment (sample size)	Size Class	Mean Charcoal mass (g)	Carbon Concentration (%)	Mean Charcoal C mass (g)	A and O horizon Charcoal C % of Total Soil C
Control (33)	>2 mm	0.3 (0.1)	55 (0.0)	0.1 (0.0)	1.4 (0.0)
	1–2 mm	0.0 (0.0)	53 (0.0)	0.0 (0.0)	
Burn Only (53)	>2 mm	1.7 (0.1)	60 (0.0)	1.0 (0.1)	0.94 (0.0)
	1–2 mm	0.7 (0.3)	54 (0.0)	0.2 (0.2)	
Understory thin/burn (89)	>2 mm	1.8 (0.4)	60 (0.0)	1.1 (0.3)	1.5 (0.0)
	1–2 mm	0.2 (0.0)	50 (0.0)	0.0 (0.0)	
Overstory thin/burn (72)	>2 mm	1.8 (0.2)	60 (0.0)	1.1 (0.1)	0.2 (0.0)
	1–2 mm	0.1 (0.0)	51 (0.0)	0.0 (0.0)	

Atomic ratios provide an indication of the aromaticity of charred material [59], where material that is more charred generally has smaller H/C and O/C ratios [60]. Our atomic ratios for H/C and O/C were higher than those reported by Nocentini et al. [47] and our O/C values were higher than those reported by Michelotti and Miesel [61], suggesting that the charcoal at our study site may be less charred than these other studies (Table 3). The difference in results may be in part a function of fire type and time since fire, as both Nocentini et al. [47] and Michelotti and Miesel [61] sampled in areas burned by wildfire two weeks and 2.5 years after fire, respectively, whereas our study was conducted 12 years after fire in areas burned by low-severity surface fire. Charcoal may experience compositional changes over time when exposed to environmental conditions, as Ascough et al. [62] demonstrated using laboratory oxidation of charcoal samples between <50 and 50,000 years of age. Because we did not collect charcoal immediately after fire, we are unable to assess the extent to which the chemical composition of our samples has been affected by biological or photo-oxidation, or adsorption of non-charred organic compounds.

**Table 3:** Mean and (standard error) of elemental concentrations, elemental mass ratios and atomic ratios for > 2mm charcoal in the A horizon only. Values in parentheses are standard deviations of the means.

Treatment	Charcoal elemental concentrations (g kg^-1^)			Elemental mass ratios			Atomic ratios		
	C	N	H	C/N	H/C	O/C	C/N	H/C	O/C
Control	541.0 (79.2)	8.0 (2.6)	33.7 (5.8)	76.9 (1.6)	0.06 (0.0)	0.76 (0.0)	89.6 (35.4)	0.71 (0.2)	0.47 (0.4)
Burn only	595.5 (65.5)	8.3 (3.3)	33.5 (4.1)	80.4 (0.6)	0.06 (0.0)	0.68 (0.0)	93.7 (39.2)	0.71 (0.1)	0.41 (0.2)
Understory thin/burn	601.6 (56.5)	9.0 (3.3)	33.4 (8.7)	73.5 (0.5)	0.06 (0.0)	0.61 (0.0)	85.7 (28.6)	0.71 (0.2)	0.36 (0.1)
Overstory thin/burn	599.2 (73.3)	8.3 (2.9)	32.2 (4.4)	81.8 (0.8)	0.05 (0.0)	0.66 (0.0)	95.3 (33.3)	0.60 (0.1)	0.40 (0.5)

Although charcoal is perceived as one of the most stable forms of organic matter in soils, charred material in soils can be heterogenous depending on the characteristics of the pre-fire biomass [61, 63] and on the charring conditions during a fire [59, 63, 64]. However, the aromaticity and stability of more broadly defined pyrogenic C (i.e., not limited to visually identified macroscopic charcoal particles) have been positively associated with heating temperature [65–67]. Stability of char in the environment is also influenced by particle size, physical location within the soil profile, and association in soil aggregates [27, 47, 68]. Michelotti and Miesel [61] showed that elemental concentrations and atomic ratios differed among char types and that the chemical composition of woody debris charred during the fire suggested a greater extent of charring relative to charred bark or charred pine cones in a stand-replacing wildfire in jack pine (*P*. *banksiana Lamb*.) forest in Michigan. While we did not differentiate between char source material for the chemical analysis, our results do provide an average characterization of the chemical composition of char in the upper 5 cm of the A horizon across different treatments (Table 3).

### Woody debris and charcoal C pool

We hypothesized that soil charcoal amount derived from CWD would be greater than that derived from FWD, but found no significant difference between CWD and FWD using linear regression (burn only A-hor: R^2^ = 0.1 p = 0.1 O-hor: R^2^ = 0.0 p = 0.7, understory thin/burn A-hor: R^2^ = 0 p = 0.8 O-hor: R^2^ = 0.0 p = 0.1, overstory thin/burn A-hor: R^2^ = 0 p = 0.9 O-hor: R^2^ = 0 p = 0.9). There are three potential causes of this finding, 1) there is in fact no difference in charcoal C pools between the different fuel size classes, 2) the charcoal produced from CWD gets distributed further from the log than the 60 cm distance we sampled, or 3) the conditions that cause char formation on CWD and in soil are similar. Charcoal pieces that were formed from the combustion of CWD and shed from the sampled log may have been transported down slope of the log either by gravity or erosion; however the timescale at which these processes may occur has not been tested. Given that MacKenzie *et al*. [69] found little to no spatial auto-correlation in charcoal content across the forest floor, this finding warrants additional investigation because if there is no differential effect of fuel size on charcoal C pools with prescribed burning, our treatment-level estimates of charcoal pools are an under-estimate of the actual charcoal produced because FWD is distributed more evenly than CWD.

**Fig 1:**
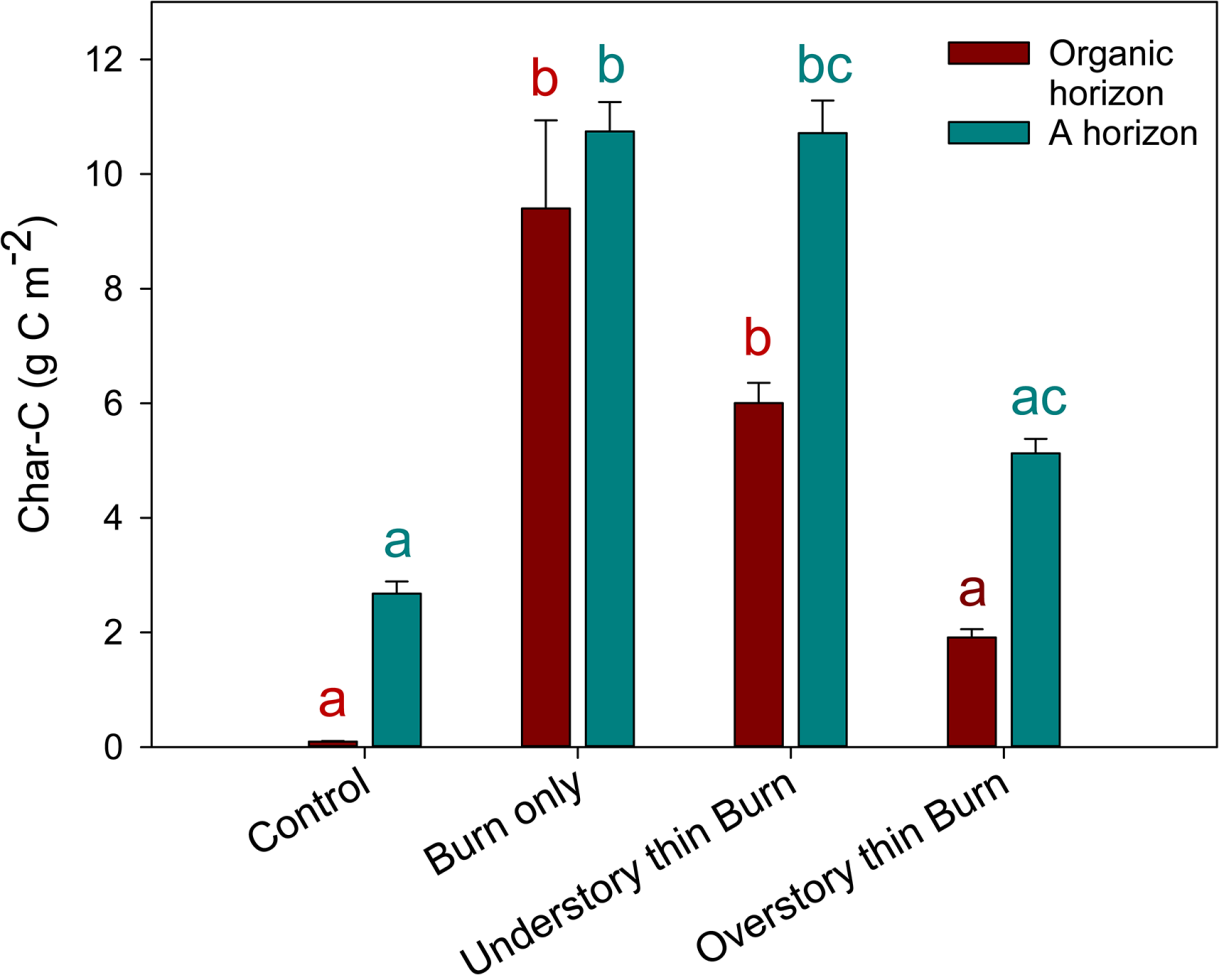
Mean charcoal C (g C m^-2^) and standard error for logs within treatments that include prescribed burning (n = 30 logs per treatment unit) and the control (n = 10 logs per treatment unit). Carbon stock comparisons were made across treatments within soil horizon. Same colored bars with different letters are significantly different (*p*≤0.05).

Since there was no effect of distance from log on charcoal amount, all samples were used to determine charcoal C pool sizes at individual logs for treatment comparisons. Charcoal_ahor_ C (10.7 and 10.7 g C m^2^, respectively) and charcoal_Ohor_ C (9.4 and 6.0 g C m^2^, respectively) of the burn-only and understory-thin and burn treatments was significantly greater than charcoal C in both the A and organic horizon (charcoal_ahor_: 2.7 g C m^2^, charcoal_Ohor_: 0.1 g C m^2^) (Fig 1) in the control. Charcoal C in the overstory-thin and burn (charcoal_ahor_: 5.12 g C m^2^, charcoal_Ohor_: 1.9 g C m^2^,) was not significantly different from the control. In the overstory thin and burn Charcoal_ahor_ C was not significantly different from the understory-thin and burn (Fig 1).

### Log abundance and charcoal C pool

**Fig 2:**
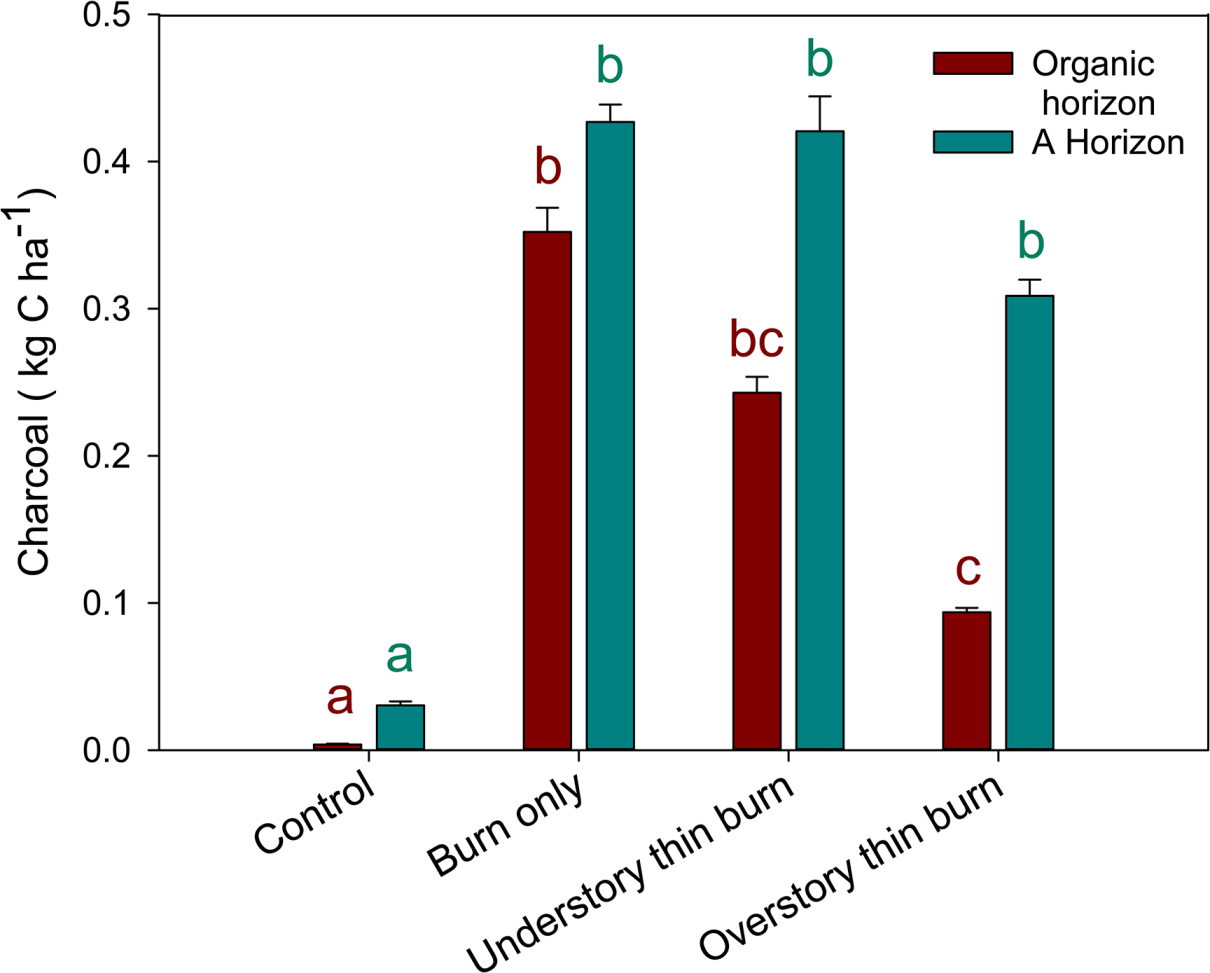
Mean carbon stock comparisons at the treatment unit level. Total charcoal carbon was estimated by scaling sampled log results to all logs in the complete coarse woody debris inventory. Mean comparisons are between the same horizon and bars with different letters are significantly different (*p*≤0.05) (n = 3).

To estimate the impact that prescribed fire had on charcoal C amount as a function of log abundance within treatments, we extrapolated the amount of charcoal formed from the combustion of logs to each four hectare treatment unit (Fig 2). Our results indicate that prescribed burning is a significant source of charcoal in this mixed-conifer forest. All treatments that included burning, in both soil horizons, had significantly more C than the control (control, charcoal_Ohor_: 3.7 g C ha^-1^, charcoal_Ahor_: 30.3 g C ha^-1^) (Fig 2). The only statistically significant differences detected between treatments that were burned were in charcoal_Ohor_ C, where the amount of charcoal C produced in the overstory-thin and burn treatment (93.7 g C ha^-1^) was lower than the burn only and understory-thin and burn treatments (352.2 g C ha^-1^, 242.8 g C ha^-1^, respectively) (Fig 2).

To determine the relative contribution of charcoal in the O horizon and top 5 cm of the A horizon to total ecosystem C within treatments, we placed the results from this research in the context of a previous study. Wiechmann *et al*. [41] found that total ecosystem C among treatments ranged from 201–441 Mg C ha^-1^, with live tree C (78–287 Mg C ha^-1^) having the largest stock. Total ecosystem C ranged from 201–398 Mg C ha^-1^ across the treatments that included prescribed burning, with live tree C (78–259 Mg C ha^-1^) having the largest stock in burn treatments [41]. Charcoal contributed 0.40–0.68% of total ecosystem C storage (Table 3). Results by treatment for organic and A horizons show significantly more charcoal in the burn only and understory-thin and burn than in the control (Fig 1). While charcoal in both the overstory-thin and burn A and organic horizon was not significantly different from the control, when scaled to the treatment level the overstory-thin and burn had a significantly greater amount of charcoal mass than the control (Fig 2).

These results demonstrate the effect of pre-fire CWD load on charcoal amount (Fig 2). North et al. [70] reported that CWD in the burn only (24.1 Mg C ha^-1^), understory-thin and burn (23.0 Mg C ha^-1^), and overstory-thin and burn (18.6 Mg C ha^-1^) was reduced by 61, 61, and 58%, respectively as a result of treatment. Post-burn charcoal mass shows a similar pattern to pre-burn CWD C reported in North et al. [68], suggesting that pre-burn fuel load may be one determinant of charcoal pool increase during burning. The other factor, for which we did not collect data that may have influenced charcoal differences between treatments is the effect of fire behavior. Canopy cover can influence microclimatic conditions [71], such as relative humidity and temperature, which influence fire behavior. Additionally, FWD fuel loads have been found to drive fire patterns [72,73]. More FWD in the form of smaller downed branches and debris that had fallen during mechanical thinning may have led to more active fire behavior, converting more surface biomass into ash or soot, as opposed to charcoal in soils in the overstory-thin and burn treatment. In a study that quantified charcoal at sites that experienced different burn severities, Pingree et al. [38] quantified the least amount of charcoal in areas that experienced the highest fire severity, temperatures, convection rates, and erosion. Similarly, Buma *et al*. [74] found that more downed overstory biomass (resulting from wind disturbance) led to higher fire intensity (increased aboveground fire temperature and duration), thereby reducing black C formation in a subalpine forest. Additionally, higher C/N ratios for wood charcoal generally result from burn conditions of higher temperature and duration [75]. Charcoal in the overstory-thin and burn treatment had the highest C/N ratio (Table 2). Data on fire behavior, such as duration, temperature, and proportion of smoldering combustion may provide additional insight into variation in charcoal pools as a function of forest structure and fuel loads.

Overall, we found more charcoal in the first 5 cm of the A horizon than in the organic horizon (Figs 1 and 2). The movement of charcoal vertically down the soil profile could be attributed to leaching of particles carried downward through suspension (lessivage) or bioturbation (reworking of soils and sediments by animals or plants). Movement of charcoal to deeper depths may lead to increased C sequestration over longer time scales, because charcoal would be protected from erosion off site or combustion by subsequent fire events, suggesting that mineral soils reflect an important pool of C influenced by the long-term input of charcoal [37]. MacKenzie *et al*. [69] found charcoal C content in Sierran mixed-conifer forests to range from 2.0–4.5 Mg C ha^-1^ in the surface 6 cm of mineral soil, and measured a similar charcoal C amount at a depth up to 60 cm. Our study quantified considerably less charcoal C in the same ecosystem (0.03–0.78 Mg C ha^-1^, Table 4). One possible explanation for the discrepancy in results could be methodological. We used visual separation to quantify charcoal and focused on macro-particles, while MacKenzie *et al*. [69] used a chemical digestion method that was not constrained to macro-particles. Additionally, the average percent C in charcoal in this study fell below the range of percent C in charcoal following wildfires found in the literature (70–80%; [21,76]). In this study (with the exception of the control) all charcoal size fractions had a C concentration of approximately 60%, similar to another prescribed burn experiment (65% C; [77]). The low C concentration (15%) from charcoal pieces in the control organic horizon may have resulted from contamination by mineral soil particles or by variations in char depth of the particles.

**Table 4:** The 2013 charcoal C stock size and the total C stock size (Mg C ha^-1^) of ecosystem C pools 12 years post-treatment for the control and three burn treatments. Treatments were conducted in 2001. All ecosystem component values, except for charcoal, obtained from Wiechmann et al. [41]. Values in parentheses are standard errors of mean.

Ecosystem component	Control	Burn only	Understory thin burn	Overstory thin burn
Live tree	287 (34)	259 (15)	170 (3.9)	78.4 (19)
Shrub	0.02 (0.0)	0.02 (0.0)	0.04 (0.0)	0.07 (0.0)
Snag	45.6 (10)	45.2 (1.3)	48.1 (8.4)	28.0 (2.5)
CWD	15.4 (1.9)	12.1 (4.4)	26.6 (6.4)	17.1 (3.6)
FWD	12.7 (1.5)	10.2 (0.8)	7.03 (2.8)	8.19 (0.9)
Organic horizon	5.20 (0.83)	4.90 (0.9)	4.83 (1.5)	2.91 (0.53)
Soil (0–30 cm)	75.2 (10)	67.3 (4.7)	84.8 (5.8)	66.7 (1.6)
Charcoal	0.03 (0.0)	0.78 (0.0)	0.66 (0.0)	0.40 (0.0)
Total	440 (39)	399 (35)	331 (22)	201 (12)
Charcoal % of total	0.01%	0.20%	0.20%	0.20%

### Predicting charcoal C

**Fig 3:**
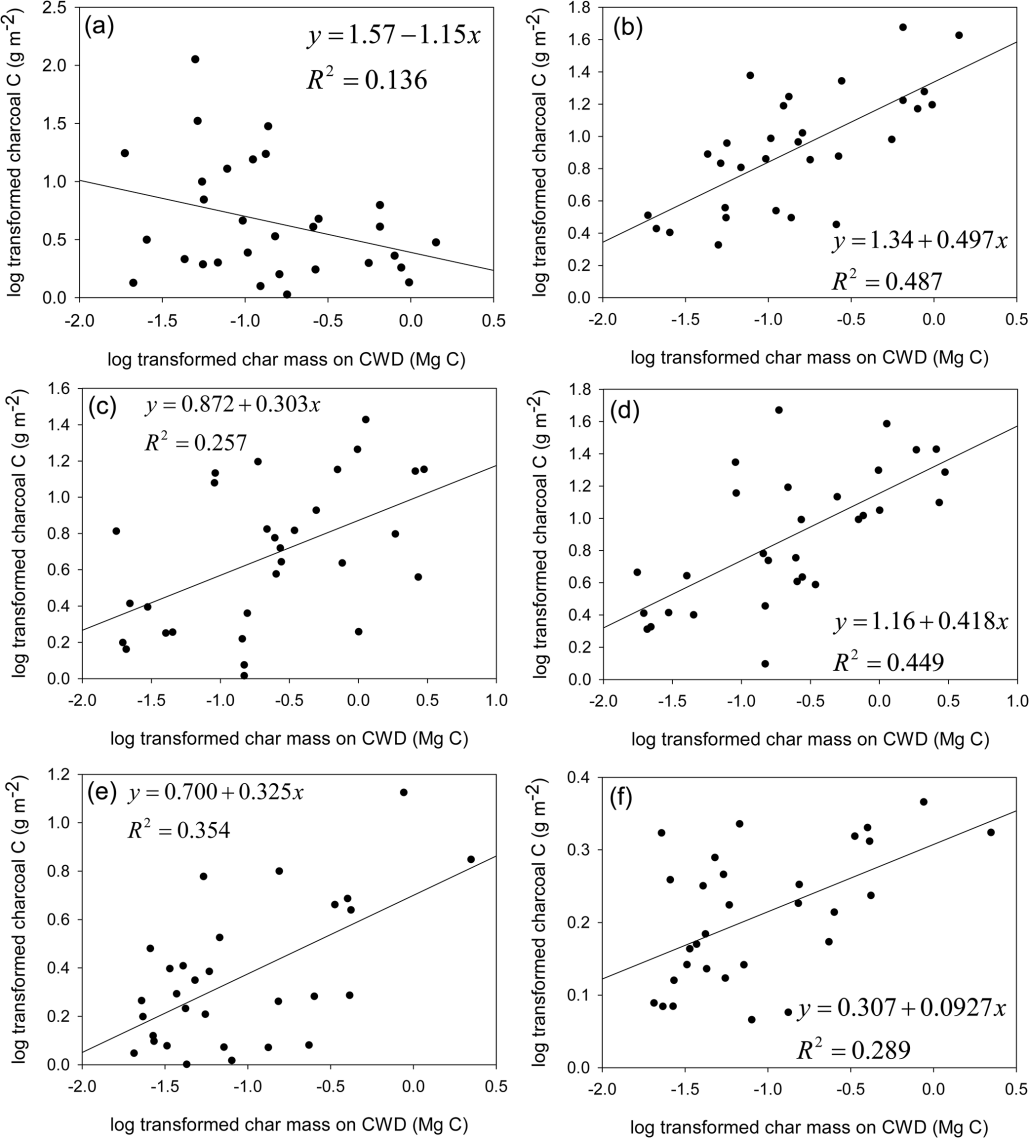
Regression results for charred mass on sampled CWD as a predictor for charcoal carbon. Figures in the left column are results for the organic horizon. Figures in the right column are results for the A horizon (0–5 cm). The first row (a-b) is results for the burn-only, the second row (c-d) is results for the understory-thin and burn, and the third row (e-f) is results for the overstory-thin and burn. Note that both x- and y-axis scales vary by panel. Both CWD char mass and charcoal mass were log transformed to meet the assumption of normality.

Of the candidate set of predictors, across treatments and soil layers, the mass of charring on log immediately after treatment (char mass on CWD) was a very influential predictor of charcoal C amount in the soil (Tables 5 and 6, Fig 3). In several cases, models with 2002 CWD % charred as the predictor were ranked as the second best model, based on AIC_C_ values (Table 5 and Table 6). However, in these cases AIC_C_ values were within two of the best model, indicating equal support. All relationships of char mass on CWD and charcoal C were positive, with the exception of charcoal_Ohor_ C amount in the burn only (Fig 3A). The negative relationship in the burn only may be the result of differences in fire behavior and intensity between unthinned and thinned plots. Previous research has found that the conditions associated with different fire types yield different charcoal production rates [22]. Char mass on CWD explained between 18 and 35% of the charcoal_Ohor_ C variance and 29–49% of the variance of the charcoal_Ahor_ C (Table 5 and Table 6). While char mass on CWD is a contributing factor to charcoal production, other predictor variables, such as those relating to fire behavior, may improve the ability to estimate charcoal production from prescribed burning. Intensity measurements such as fire-line intensity may help better predict charcoal formation; oxygen availability and fire duration have also been cited as possible causes of differences in charcoal production [22]. In addition, fire temperature may also be an important charcoal formation variable, because of its direct effect on charcoal structure and reflectivity [77]. Licata and Sanford [78] reported that the most important influences on soil charcoal C formation are available biomass sources and the fire regime. Lignin-based surface fuels (woody debris) and deep organic horizons are two large charcoal C sources that result in relatively higher charcoal C per fire [78]. Although models that included litter depth were not among the best models to predict charcoal C in this study, pre-fire litter depth was, on average, greatest in the burn only treatments (1.75 cm) followed by the understory-thin and burn (1.3 cm) and overstory-thin and burn litter depths (0.58 cm). Given the variability in charcoal pools between the treatments, predictor variables relating to fire behavior may improve the ability to estimate charcoal pools. Although our results provide a field-based estimate of charcoal C, obtaining a mechanistic understanding of the relationship between biomass amount and type, fire behavior, and charcoal production may require a laboratory-based study that tightly controls variability in these factors.

**Table 5:** Two best-fit models for each treatment with the smallest AIC_C_ values for modeling charcoal_Ohor_ C (g C m^-2^). Δ AIC_C_ is the difference in AIC values from the model with the lowest AIC_C_.

Treatment	Predictor(s)	AIC_C_	Δ AIC_C_	R^2^
Burn only	Fine woody debris (Mg C ha^-1^)	5.5	-	0.14
	Char mass on CWD (Mg C)	5.6	0.0	0.18
	Fine woody debris (Mg C ha^-1^) + 2002 percent ash	9.8	4.3	0.14
Understory thin and burn	Fine woody debris (Mg C ha^-1^)	5.8	-	0.09
	Char mass on CWD (Mg C)	6.0	0.2	0.26
Overstory thin and burn	Char mass on CWD (Mg C)	6.7	-	0.35
	2002 CWD charred (%) + Char mass on CWD (Mg C)	11	4.3	0.37

**Table 6:** Two best-fit models for each treatment with the smallest AIC_C_ values for modeling charcoal_Ahor_ C (g C m^-2^). Δ AIC_C_ is the difference in AIC values from the model with the lowest AIC_C_.

Treatment	Predictor(s)	AIC_C_	Δ AIC_C_	R^2^
Burn only	2002 CWD charred (%)	6.1	-	0.16
	Char mass on CWD (Mg C)	6.5	0.0	0.49
Understory thin and burn	Char mass on CWD (Mg C)	6.2	-	0.45
	2002 CWD charred (%) + percent ash	10	3.9	0.12
Overstory thin and burn	Percent ash	8.6	-	0.25
	Char mass on CWD (Mg C)	8.6	0.1	0.29

Our results demonstrate that prescribed burning significantly increases the charcoal pool relative to the control. But, uncertainty remains regarding a mechanistic understanding of charcoal production and subsequent chemical properties, which warrants further investigation. A controlled burning experiment to isolate the effects of changes in parameters that alter fire behavior would be beneficial for better understanding the charcoal formation process as it relates to variables that can be field-measured. Improving our understanding of the effects of restoring natural fire regimes on charcoal C will improve our ability to quantify the effects of restoration treatments on forest C dynamics.

## Supporting Information

S1 Fig The soil core sampling layout at each log. The lettered blue x’s symbolize different sampling sites, where organic (Ohor) and the top 5cm of the A horizon (Ahor) were cored. The black x’s located on the log represent sample locations of charring depth on the log.
(PDF)
