# Supplementary material for: Macro-Particle Charcoal C Content following Prescribed Burning in a Mixed-Conifer Forest, Sierra Nevada, California
Source: PLoS One. 2015 Aug 10;10(8):e0135014. doi: 10.1371/journal.pone.0135014 (PMC4530888; doi:10.1371/journal.pone.0135014)
Supplement: S1 Fig — The lettered blue x’s symbolize different sampling sites, where organic (Ohor) and the top 5cm of the A horizon (Ahor) were cored. The black x’s located on the log represent sample locations of charring depth on the log. (PDF) [file pone.0135014.s001.pdf]

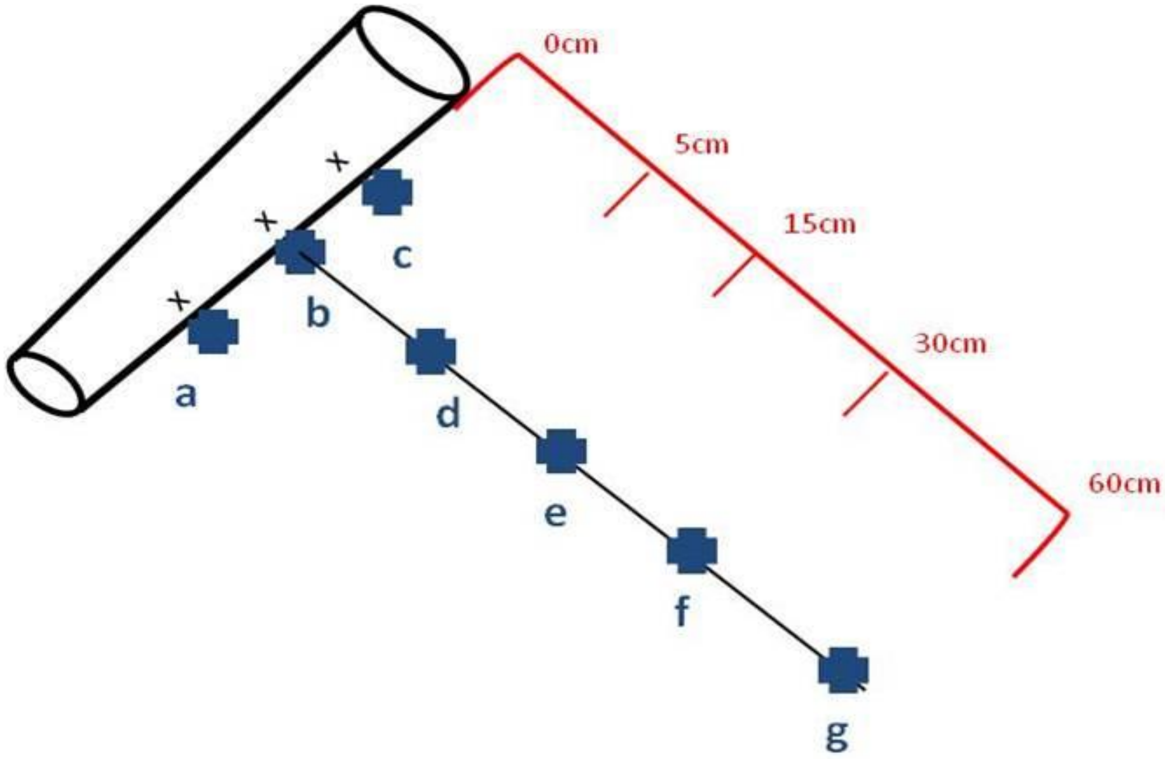

S1 Figure. The soil core sampling layout at each log. The lettered blue x's symbolize different sampling sites, where organic (Ohor) and the top 5cm of the A horizon (Ahor) were cored. The black x's located on the log represent sample locations of charring depth on the log.
